# Exercise and Physical Activity Levels and Associated Factors Among High-Risk Pregnant Women

**DOI:** 10.1055/s-0042-1743099

**Published:** 2022-03-11

**Authors:** Larissa Antunes Miranda, Anna Caroline Ribeiro de Moura, Karina Tamy Kasawara, Fernanda Garanhani Surita, Mayle Andrade Moreira, Simony Lira do Nascimento

**Affiliations:** 1 Department of Physical Therapy, Federal University Ceará, Fortaleza, CE, Brazil; 2 Department of Physical Therapy, University of Toronto, Toronto, Canada; 3 Department of Obstetrics and Gynecology, Universidade Estadual de Campinas, Campinas, SP, Brazil

**Keywords:** pregnancy, high-risk pregnancies, exercise, sedentary behavior, motor activity, gravidez, gravidez de alto risco, exercício físico, atividade motora, comportamento sedentário

## Abstract

**Objective** To assess the levels of physical activity and exercise practice, and examine the associated maternal characteristics; as well as the anxiety levels of high-risk pregnant women.

**Methods** A cross-sectional study conducted with pregnant women at a High-risk Prenatal Clinic (HRPC) in a tertiary maternity. Pregnant women of 18 to 40-years-old, with a single fetus, and with gestational age up to 38 weeks were included. The level of physical activity and exercise practice of the study's participants were investigated using the Pregnancy Physical Activity Questionnaire (PPAQ). Maternal sociodemographic, anthropometric, and medical data were investigated using a specific form. For anxiety levels, the short version of the State-Trait Anxiety Inventory (STAI) was applied. We used the Student *t* -test, chi-square test, odds ratio (OR) with 95% confidence interval (95% CI) and multiple logistic regression. The significance level was 5%.

**Results** Among the 109 pregnant women included, 82 (75.2%) were classified as sedentary/little active. The higher energy expenditure were for domestic activities (133.81 ± 81.84 METs), followed by work-related activities (40.77 ± 84.71 METs). Only 19.3% women exercised during pregnancy (4.76 ± 12.47 METs), with slow walking being the most reported exercise. A higher level of education was the most important factor associated with women being moderately or vigorously active (OR = 29.8; 95% CI 4.9–117.8). Nulliparity (OR = 3.1; 95% CI 1.0–9.1), low levels of anxiety (OR = 3.6; 95% CI 1.2–10.7), and unemployment (OR = 4.8; 95% CI 1.1–19.6) were associated with the practice of exercise during pregnancy.

**Conclusion** Most women with high-risk pregnancies exhibited a sedentary pattern, with low prevalence of physical exercise practice. Recognizing factors that hinder the adoption of a more physically active lifestyle is essential for an individualized guidance regarding exercise during pregnancy.

## Resumo

**Objetivo** Analisar o nível de atividade física e a prática de exercício físico, examinar as características maternas associadas, assim como níveis de ansiedade de gestantes de alto risco.

**Métodos** Estudo observacional, transversal e quantitativo, realizado no ambulatório de Pré-Natal de Alto Risco (PNAR) de uma maternidade terciária. Foram incluídas gestantes com idade entre de 18 e 40 anos; feto único e com idade gestacional (IG) até 38 semanas. O nível de atividade física e prática de exercício físico das participantes do estudo foram investigados usando o Questionário de Atividade Física na Gestação (QAFG). Os dados sociodemográficos, antropométricos e médicos maternais foram investigados usando um formulário específico. Para níveis de ansiedade, a versão curta do Inventário de Ansiedade Traço-Estado (STAI) foi aplicado. Usamos o teste t de Student, teste qui-quadrado, razão de chances (OR) com um intervalo de confiança de 95% (IC 95%) e regressão logística multinomial. O nível de significância considerado foi 5%.

**Resultados** Das 109 gestantes incluídas no estudo, 82 (75,2%) foi classificada como sedentária/pouco ativa. Os maiores gastos energéticos foram em atividades domésticas (133.81 ± 81.84 METs), seguidas pelas atividades ocupacionais (40.77 ± 84.71 METs). Apenas 19.3% praticaram exercício físico durante a gravidez (4.76 ± 12.47 METs), sendo a caminhada lenta a atividade mais relatada. Maior grau de escolaridade foi o fator mais importante associado a gestante ser moderadamente ou vigorosamente ativa (OR = 29,8; IC 95% 4,9–117,8) . Nuliparidade (OR = 3,1; IC 95% 1,0–9,1), baixos níveis de ansiedade (OR = 3,6; IC 95% 1,2–10,7) e não trabalhar na gestação (OR = 4,8; IC 95% 1.1–19,6) foram associados à prática de exercício físico durante a gestação.

**Conclusão** A maioria das gestantes de alto risco desenvolveram um padrão sedentário, com baixa prevalência da prática de exercício físico. Reconhecer os fatores que dificultam a adoção de um estilo de vida mais ativo fisicamente é fundamental para a orientação adequada e individualizada acerca da prática de exercício físico durante a gestação.

## Introduction

In 1985, a guideline with recommendations regarding physical activity during pregnancy was published for the first time by the American College of Obstetricians and Gynecologists (ACOG), though they are now considered conservative. Since then, the practice of physical activity and exercise during pregnancy has gained notoriety, due to its potential benefits to maternal and fetal health. 1 2 3 4

Given this context, it is essential to properly differentiate the concepts of physical activity and exercise. According to Caspersen et al., 5 physical activity is related to any body movements performed by skeletal muscles, in which energy expenditure is above basal, such as labor, domestic, and leisure activities. On the other hand, exercise is defined as a planned, structured and repetitive physical activity, which is desired to achieve improvement or maintenance of physical fitness.

Although the numerous maternal and fetal health benefits of an active pregnancy are recognized, several studies in Brazil demonstrate alarming data on sedentary behavior among women with habitual risk pregnancies, and the restriction of physical activities is even more reinforced for high-risk pregnant women. 6 7 8 9 10

The evaluation of energy expenditure and knowledge of the physical activities in which pregnant women participate allows for a better understanding of the women's profile and an adequate exercise prescription by health professionals. In this context, the application of questionnaires to measure the level of physical activity and exercise practice is a valid and useful tool, in the absence of more objective methods, such as direct or indirect calorimetry, accelerometers, and electronic movement sensors. The Pregnant Physical Activity Questionnaire (PPAQ) has been proven to be effective, since it addresses activities which are frequently present in the daily lives of pregnant women, such as domestic, sports, and work-related activities. Thus, it can be applied to women with low and high-risk pregnancies. 9 11 12

Several conditions can make a pregnancy high risk, including biological factors (health conditions, chronic diseases, mother's age, and nutritional and genetic aspects), psychosocial (lifestyle, emotional disturbance, and relationships), social aspects (prenatal negligence and social vulnerability), and clinical or obstetric complications that happen during pregnancy. Thus, high-risk pregnancy is defined here as any medical or obstetric conditions associated with a pregnancy with an actual or potential hazard to the health or well-being of the mother or fetus that requires specialized care. 13 14 15

Chronic diseases such as diabetes mellitus and arterial hypertension, as well as overweight and obesity during pregnancy are described as contributing factors to the low adherence of women to activities with higher energy expenditure. Other reasons that also contribute to a less active lifestyle during pregnancy are: lack of infrastructure (for example, parks and places to walk), number of children at home, other occupations that limit time, little family incentive, perception of safety in public spaces. Furthermore, psychological changes, such as anxiety and depression, can be barriers capable of hindering practice of physical activities and exercise. Among sociodemographic factors, lower educational levels and income, and higher number of children at home are most frequently associated with reduced physical activity. 6 16 17

As sedentary behaviors seem to be even more reinforced to high-risk pregnant women, we hypothesized that women with high-risk pregnancies would have a sedentary profile. Thus, this study aimed to assess the physical activity and exercise practice levels of high-risk pregnant women and to examine the maternal characteristics associated with exercising and level of physical activity during pregnancy. 7

## Methods

This is a cross-sectional study involving pregnant women attended at Maternal Fetal Medicine Service (MFMS) and High-Risk Prenatal Clinic (HRPC) of the Maternity Hospital School Assis Chateaubriand (MEAC) of the Federal University of Ceará, a reference center in maternal and childcare, in Fortaleza, Ceará, Brazil. Our facility provides care for women and newborns from the least favored segment of the population of Ceará and the Northeastern region. Data collection was performed from August 2017 to July 2019.

Eligibility criteria were pregnant women between 18 and 40 years old, single fetus, gestational age up to 38 weeks, who were attended in the service at the HRPC. High-risk pregnancy is defined as any medical or obstetric condition related to pregnancy, with an actual or potential hazard to the health or well-being of the mother or fetus, and requires specialized care. 13 14 15 Pregnant women with absolute contraindication to perform physical activity during pregnancy according to ACOG criteria were excluded (hemodynamically significant heart disease, restrictive lumbar disease, incompetent cervix or cerclage, multiple pregnancy at risk of premature birth, premature labor during the current pregnancy, rupture of membranes, pre-eclampsia, and severe anemia). 18

During antenatal care (ANC) visits, women who met the eligibility criteria were invited to participate of study. Following consent, women were interviewed using standardized questionnaires: a questionnaire developed by the researchers regarding patients' socioeconomic status (age, self-reported skin color/race, marital status, educational degree, monthly family income, employment status), anthropometric data (weight, height, and pre-pregnancy body mass index [BMI]), the State-Trait Anxiety Inventory (STAI) short version, and additional data on participants' obstetric history, such as parity, gestational age, and pregnancy comorbidities were collected from medical records and prenatal care cards—independent variables. Then, all patients answered a questionnaire on physical activities, including daily amount of physical activity and exercise practice, specifically for pregnant women—dependent variables.

We assumed physical activity was any voluntary, corporal movement that increased the metabolism above its resting rate, such as labor, domestic, and leisure activities. And exercise was defined as structured, planned, and repetitive physical activity intended to promote health and maintain one or more components of physical ability. 5

For both outcomes, this study used the PPAQ validated for Brazilian Portuguese. The PPAQ requests respondents to report the time spent participating in 31 activities including household (5 activities), caregiving (6 activities), occupational (5 activities), sports/exercise (9 activities), transportation (3 activities), and inactivity (3 activities). 17 18

For the classification of the level of physical activity, the calculation of the energy expenditure in the metabolic equivalent of task (MET) was performed for each domain of physical activity (locomotion, leisure, domestic and occupational activities) based on the type, duration, and frequency of physical activity. The total daily energy expenditure, used to classify the pregnant woman in levels of physical activity (sedentary, little active, moderately active, and vigorously active), was calculated according to the FAO/WHO/UNU (2001) criteria.

In this calculation, it is considered that the minimum expenditure of the subjects is equal to their baseline, that is, a MET multiplied by 24 hours (MET-h). The level of physical activity is considered the total energy expenditure expressed as a multiple of the daily basal metabolic rate, based on the ratio: calculated total daily MET / 24 MET. For this reason, we categorized the level of physical activity of the pregnant woman as following: sedentary / little active (≤ 1.69), moderately active (1.70–1.99) and vigorously active (> 2.00). For the analysis, we categorized women in little active versus moderately and vigorously active. 19

The prevalence of exercise was assessed using questions 18 to 26 of the PPAQ (sports/exercise), referring to different types of exercise.

To classify the level of anxiety, the short version of the State-Trait Anxiety Inventory (STAI) validated for the Brazilian Portuguese was applied. To calculate the total STAI score (range 20–80) the reverse score of the positive items (calm, at ease, and relaxed) was added to the score of the negative items (tense, nervous, and worried), the result was then multiplied by 20/6. Following the classification established by Araújo et al., 20 women with a score ≤ 40 were classified as having a low level of anxiety, and those with a score greater than 40 with a high level. 20 21 22

To minimize information bias, all data were collected in a private environment through a standardized interview by previously trained evaluators.

The data were analyzed using the Statistical Package Social Sciences (SPSS, IBM Corp. Armonk, NY, USA) software. Continuous variables are presented as mean (M) and standard deviation (SD), and categorical outcomes in absolute and relative frequencies. To identify the factors associated with the level of physical activity (categorized in sedentary/little vs. moderately/vigorously) and the practice of exercise (sedentary vs. active), either the Chi-square or Fisher exact tests were performed, followed by a multiple logistic regression. Odds ratio (OR) with 95% confidence interval (95% CI) is present for regression. The level of significance adopted was 5%.

This study was approved by the Research Ethics Committee of the Federal University of Ceará - CEP / UFC / PROPESQ number 2.474.018 (CAAE; 62916616.0.2002.5050). All participants signed a consent form confirming their agreement to participate and received a copy of it signed by the main researcher.

## Results

Of the 148 pregnant women screened at the high-risk prenatal center, 111 met the inclusion criteria, but 2 did not accept to participate in the research, thus constituting a sample of 109 participants. Of the 37 excluded pregnant women, 14 had an absolute contraindication to the practice of physical activity due to preterm labor, cervical incompetence, and vaginal bleeding, 9 were not aged between 18 and 40 years old, and the others had a gestational age greater than 38 weeks.

The analysis of participants' demographic characteristics showed an average age of 29.5 years (±5.66), most of the participants referred to themselves as brown (87.2%), had studied up to a high school educational level (47.7%), and were residing with a partner (93.5%). More than 60% of the pregnant women did not work during pregnancy and had low monthly income of approximately a minimum wage or less. Regarding the anthropometric, obstetric, and gynecological profile of the patients, the majority was classified as overweight (32.1%) or obese (36.7%). They were also predominantly multiparous (70.6%) with a mean of 28.8 ± 6.8 weeks of pregnancy. Diabetes mellitus (38%) and hypertensive syndromes (32.4%) were the most prevalent comorbidities in the current pregnancy.

The results of the PPAQ demonstrated that the pregnant women in this study had a higher average energy expenditure in domestic activities and lower expenditure in exercise practice. Regarding the METs results related to the intensity classification of the activities performed, there is a greater predominance of energy expenditure in light and sedentary activities practiced by pregnant women, with lower average energy expenditure in moderate and vigorous activities. Thus, 75.2% of our sample were considered sedentary/not very active. The prevalence of exercise practice during pregnancy was 19.3%, with slow walking being the most reported activity ( Table 1 ).

**Table 1:** Description of physical activity in MET-h/week and prevalence of exercise during pregnancy, based on the PPAQ

Physical activity end exercise variables	( *n* = 109)
**Physical activity intensity (MET-h/week)** **Sedentary**	Mean ± SD 64.81 ± 34.55
Mild	127.38 ± 63.85
Moderate	37.60 ± 51.38
Vigorous	1.15 ± 5.41
**Type of activity (MET-h/week)**	Mean ± SD
Sports/exercise	4.76 ± 12.47
Occupational	40.77 ± 84.71
Household/caregiving	133.81 ± 81.84
**Physical activity level classification**	N (%)
Sedentary/Slightly active	82 (75.2%)
Moderately active	10 (9.2%)
Vigorously active	17 (15.6%)
Prevalence of physical exercise	21 (19.3%)
**Type of physical exercise**	
Slow walk for leisure	19 (17.4%)
Quick walk for leisure	5 (4.6%)
Other activities*	8 (7.3%)

Abbreviations: MET-h, metabolic equivalent of task per hour; SD, standard deviation. Notes: * such as dancing, stretching, and squatting.

The analysis of anxiety levels according to the STAI-6 questionnaire shows the mean level of anxiety was 47.61 ± 12.63. Most of the pregnant women ( *n* = 72; 66.1%) had a total score greater than 40, showing high levels of anxiety, while only 33.9% of the women were classified as having a low level of anxiety. Among the analyzed factors related to the level of physical activity reached by patients, bivariate analysis showed that higher education level, employment, and an adequate pre-pregnancy BMI were associated with higher performance of activities with greater energy expenditure ( *p* < 0.05) ( Table 2 ). However, on logistic regression only the higher education level factor remained significative (OR = 29.8; 95% CI 4.9–177.8) ( *p* < 0.001) ( Table 3 ).

**Table 2:** Bivariate analysis of association between maternal characteristics and symptoms of anxiety and the classification of the level of physical activity among high-risk pregnant women

Variables	Sedentary/little active N = 82	Moderately/ vigorously active N = 27	*p* -value
**Age – n (%)**			
18–34 years	63 (76.8%)	19 (70.4%)	0.5
≥ 35 years	19 (23.2%)	8 (29.6%)	
**Skin color/race**			
White	8 (9.8%)	6 (22.2%)	0.093
Brown or black	74 (90.2%)	21 (77.8%)	
**Lives with a partner**			
Yes	78 (95.1%)	24 (88.9%)	
No	4 (4.9%)	3 (11.1%)	0.252
** Monthly family income ^1^**			
≤1	52 (65.0%)	13 (48.1%)	0.121
≥2	28 (35.0%)	14 (51.9%)	
**Educational level**			
Elementary school/illiterate	37 (45.1%)	4 (14.8%)	< 0.001
High school	40 (48.8%)	12 (44.4%)	
College/University	5 (6.1%)	11 (40.7%)	
**Employed**			
Yes	22 (26.8%)	15 (55.6%)	0.006
No	60 (73.2%)	12 (44.4%)	
**Parity**			
Nulliparous	24 (29.3%)	8 (29.6%)	0.971
Multiparous	58 (70.7%)	19 (70.4%)	
**Gestational trimester**			
2°	30 (36.6%)	9 (33.3%)	0.760
3°	52 (63.4%)	18 (66,7%)	
** Hypertension ^2^**			
Yes	32 (39.5%)	9 (33.3%)	0.567
No	49 (60.5%)	18 (66.7%)	
** Diabetes ^3^**			
Yes	26 (32.1%)	9 (33.3%)	0.906
No	55 (67.9%)	18 (66.6%)	
**Pre-gestational BMI**			
Normal	21 (25.6%)	13 (48.1%)	0.028
Overweight/Obesity	61 (74.4%)	14 (21.9%)	
**Anxiety**			
Low	26 (31.7%)	11 (40.7%)	0.390
Elevated	56 (68.3%)	16 (59.3%)	

Abbreviation: BMI, body mass index. Notes: ^1^ in minimum wages, ^2^ chronic or gestational hypertension, ^3^ type 2 or gestational diabetes

**Table 3:** Logistic regression model results for odds of higher level of physical activity during pregnancy according to maternal characteristics

Variable	OR	95% CI	*p* -value
Higher level of education	29.811	4.997–177.856	**<0.001**
Unemployment	0.541	0.186–1.575	0.260
Nulliparity	0.360	0.093–1.391	0.138
Adequate BMI	2.878	0.974–8.505	0.056

Abbreviations: 95% CI, 95% confidence interval; BMI, body mass index; OR, odds ratio. Notes: Independent variables included in the final logistic regression model: age (18–34 vs. ≥ 35 years); employment status (employed vs. unemployed); educational level (college and university vs. elementary or high school); family income (1 vs. 2 wage); parity (nulliparity vs. multiparity); trimester of pregnancy (2° vs. 3°); BMI (adequate vs. overweight and obesity), anxiety level (low or elevated).

The bivariate analysis ( Table 4 ) and logistic regression model ( Table 5 ) for exercise as the outcome showed that not working during pregnancy (OR = 4.8; 95% CI 1.1–19.6), nulliparity (OR = 3.1; 95% CI 1.05–9.1), and low levels of anxiety (OR = 3.6; 95% CI 1.2–10.7) were associated with exercise throughout pregnancy ( *p* < 0.05) ( Table 4 ).

**Table 4:** Bivariate analysis of association between maternal characteristics and anxiety symptoms, and exercise practice among high-risk pregnant women

Variables	Active N = 21	Sedentary N = 88	*p* -value
**Age – n (%)**			0.585
18 to 34 years	17 (81.0%)	65 (73.9%)	
≥35 years	4 (19.0%)	23 (26.1%)	
**Skin color/race**			0.297
White	1 (4.8%)	13 (14.8%)	
Brown or black	20 (95.2%)	75 (85.2%)	
**Lives with a partner**			1.0
Yes	20 (95.2%)	82 (93.2%)	
No	1 (4.8%)	6 (6.8%)	
** Monthly family income ^1^**			0.324
≤1	15 (71.4%)	60 (58.1%)	
≥2	6 (28.6%)	36 (41.9%)	
**Educational level**			0.802
Elementary school/illiterate	9 (42.9%)	32 (36.4%)	
High School	10 (47.6%)	42 (47.7%)	
College/University	2 (9.5%)	14 (15.9%)	
**Employed**			0.027
Yes	3 (14.3%)	34 (38.6%)	
No	18 (85.7%)	54 (61.4%)	
**Parity**			0.041
Nulliparous	10 (47.6%)	22 (25.0%)	
Multiparous	11 (52.4%)	66 (75.0%)	
**Gestational trimester**			0.311
2°	5 (23.8%)	34 (38.6%)	
3°	16 (76.2%)	54 (61.4%)	
** Hypertension ^2^**			0.457
Yes	6 (30.0%)	35 (39.8%)	
No	14 (70.0%)	53 (60.2%)	
** Diabetes ^3^**			0.437
Yes	8 (40.0%)	27 (30.7%)	
No	12 (60.0%)	61 (69.3%)	
**Pre-gestational BMI**			0.565
Low weight	0 (0%)	4 (4.5%)	
Normal	8 (38.1%)	22 (25%)	
Overweight	7 (33.3%)	28 (31.8%)	
Obesity	6 (28.6%)	34 (38.6%)	
**Nutritional status**			0.218
Low weight	1 (4.8%)	2 (2.3%)	
Normal	7 (33.3%)	21 (23.9%)	
Overweight	7 (33.3%)	20 (22.7%)	
Obesity	6 (11.8%)	45 (88.2%)	
**Anxiety**			0.044
Low	11 (52.4%)	23 (29.5%)	
Elevated	10 (47.6%)	62 (70.5%)	

Abbreviation: BMI, body mass index. Notes: ^1^ in minimum wages; ^2^ chronic or gestational hypertension; ^3^ type 2 or gestational diabetes.

**Table 5:** Logistic regression model results for odds of exercising during pregnancy according to maternal characteristics

Variable	OR	95% CI	*p* -valor
Unemployment	4.811	1.180–19.620	0.028
Nulliparity	3.105	1.054–9.147	0.040
Second trimester of pregnancy	0.501	0.151–1.664	0.259
Adequate BMI	1.702	0.561–5.165	0.348
Lower level of anxiety	3.641	1.233–10.748	0.019

Abbreviations: 95% CI, 95% confidence interval; BMI, body mass index; OR, odds ratio. Independent variables included in the final logistic regression model: employment status (employed vs. unemployed); educational level (college and university vs. elementary or high school); family income (1 vs. 2 wage); parity (nulliparity vs. multiparity); trimester of pregnancy (2° vs. 3°); BMI (adequate vs. overweight and obesity), anxiety level (low or elevated).

## Discussion

The findings of this study show that high-risk pregnant women have a preferentially sedentary lifestyle, with a predominance of energy expenditure in domestic activities. Furthermore, the prevalence of exercise during pregnancy is quite low, with only 19.3% of this study's participants reporting some practice. Slow walking was the most common type of activity. The factors associated with higher levels of physical activity are working during pregnancy and higher level of education, whereas the factors associated with exercising during pregnancy are nulliparity, unemployment, and a low level of anxiety.

Studies conducted in different countries, including Brazil, reveal that women tend to reduce the level, intensity, and duration of exercise during pregnancy, corroborating the findings presented in this article. Other studies of our research group in Southern Brazil found an even lower prevalence than the one found in the present research: 14.8% of women were active before pregnancy and 12.9% during pregnancy. The prevalence decreased throughout pregnancy, and only 4.3% of the participants remained active until the end of the pregnancy. 9 23 24 25 To assess the physical activity levels, Silva 19 validated the PPAQ with pregnant Brazilian women and found that 80% of the women either performed mild-intensity activities or were sedentary, and that the mild-intensity activities tended to increase during pregnancy, whereas the moderate activities decreased.

It is noteworthy that we did not find studies conducted entirely with pregnant women in high-risk prenatal care. We have hypothesized that the recommendations for absolute and relative rest are further reinforced for pregnant women with comorbidities. Despite the fact that the practice of exercise plays a fundamental role in the prevention of comorbidities associated with a sedentary lifestyle, such as diabetes mellitus and arterial hypertension; it also prevents excess weight gain and postpartum weight retention. Recent guidelines make clear the absolute contraindication to exercise during pregnancy such as: ruptured membranes, premature labour, unexplained persistent vaginal bleeding, placenta praevia after 28 weeks’ gestation, pre-eclampsia, incompetent cervix, intrauterine growth restriction, high-order multiple pregnancy (eg, triplets), uncontrolled type I diabetes, uncontrolled hypertension, uncontrolled thyroid disease, other serious cardiovascular, respiratory or systemic disorder. 1 6

Several studies have showed walking is the most common type of physical activity practiced by pregnant women. Walking becomes the most accessible exercise, since it is easily integrated into the daily routine, and it does not require equipment or payment to be performed. Another possible reason is the traditional belief that walking during pregnancy is safe and can make delivery easier. However, in addition to aerobics, the more recent guidelines recommend the practice of strength exercises during pregnancy. 1 6 26 27

Many studies seek to understand which are the barriers and the facilitators for the practice of exercises and for a higher level of physical activity amidst pregnant women. The present study shows that pregnant women with a higher educational level are associated with higher energy expenditure. Women with low educational level seem to have beliefs related to poor diets and physical inactivity, such as: activities associated with daily life can replace activities with greater energy expenditure. 16 26 27 28 29 30

Regarding parity, a systematic literature review identified that pregnant women with at least one child tend to interrupt the practice of sports and exercises during pregnancy when compared with women who do not have children. Nevertheless, while pregnant women who already have one or more children report less time to exercise, they have a higher overall energy expenditure due to the increased activities of daily living, such as playing with children and household activities. 17 Still, some pointed out that women who work outside the house during pregnancy tend to have greater purchasing power, which reflects in healthier behaviors, such as choosing more nutritious foods and maintaining the frequency of exercise. Other studies showed that women who are not employed are more likely to comply with exercise guidelines compared with employed women. A possible explanation for this situation is the greater time availability that unemployed women have, hence including the practice of exercises into their daily routine more easily, as we found in our population. 8 18 27 29 30 31

Maternal BMI has also been associated with physical activity when compared with pre-pregnancy levels. A multicenter cohort study revealed that pregnant women with a BMI greater than 25 kg/m ^2^ cease moderate to intense physical activity during pregnancy more frequently than women with a “normal” weight (BMI 18.5–24.99 kg/m ^2^ ), who continued with moderate to vigorous activities. A possible explanation for obese and overweight pregnant women involved in less strenuous activities is negative body image and lower self-efficacy in relation to physical activities with higher energy expenditure. 16 18 24 25 31 32 33 34

Another factor that we studied was maternal anxiety. The study performed by Araújo et al. 20 with pregnant women in Rio de Janeiro found a similar result to the present study: 64.9% of these women had high levels of anxiety when answering the STAI questionnaire. 20 35 36 37 Two systematic reviews revealed low evidence about the effect of exercise on reducing anxiety symptoms during pregnancy. However, pregnant women who experience higher levels of anxiety tend to reduce self-care and have low adherence to healthy lifestyle habits, therefore, choosing more caloric foods and not exercising. Thus, anxiety symptoms should be routinely screened in pregnant women, mainly those with risk-pregnancies, to help women deal with pregnant related issues. 32 35 36 38

Our results highlight the need for a multidisciplinary approach during pre-natal care to reinforce the adoption of health-related behaviors during pregnancy, since modifiable factors such as mental health, nutritional status and physical activity patterns have been associated with better perinatal outcomes, which is the main goal of a pre-natal service. 39

As a limitation, the present study was carried in a single reference center with women from low socioeconomic level, which prevents us from generalizing our results for all high-risk pregnant women in Brazil. We used a questionnaire to assess the pattern of activity and exercise, which can generate an information bias. However, the questionnaire is a validated one, and it is also widely applied in national and international studies. Another limitation is the short period of time covered by the questionnaire which is, in this case, of three months; such period is insufficient to cover the entire pregnancy, leading to the need for longitudinal studies. However, this is the first study that includes only high-risk pregnant women evaluating both physical activity and exercise practice levels, and presenting a robust analysis of related factors. Thus, it brings some advance to the knowledge of this theme. 8 9 10 12

## Conclusion

It is observed that high-risk pregnant women adopt preferentially sedentary activities, with a predominance of energy expenditure in domestic activities. Also, the practice of exercise is greatly reduced among high-risk pregnant women. It is also noted that a higher level of education was the most important factor associated with practice of activities with greater energy expenditure. Additionally, nulliparity, unemployment, and low levels of anxiety were associated with the practice of exercise.

In view of our findings, it is plausible to acknowledge that the reduction in the practice of physical activities and exercise throughout the gestational period is a reality that involves pregnant women at habitual risk and those at high-risk. Considering the recognized benefits of exercise on maternal and fetal health, a multidisciplinary team should be engaged in prenatal care to encourage pregnant women to practice exercise properly and safely. Therefore, recognizing the factors that facilitate and hinder the adoption of a more physically active lifestyle is fundamental for an individualized orientation, manly for high-risk pregnant women.
